# RNA-seq Transcriptome Analysis of *Panax japonicus*, and Its Comparison with Other *Panax* Species to Identify Potential Genes Involved in the Saponins Biosynthesis

**DOI:** 10.3389/fpls.2016.00481

**Published:** 2016-04-12

**Authors:** Amit Rai, Mami Yamazaki, Hiroki Takahashi, Michimi Nakamura, Mareshige Kojoma, Hideyuki Suzuki, Kazuki Saito

**Affiliations:** ^1^Graduate School of Pharmaceutical Sciences, Chiba UniversityChiba, Japan; ^2^Medical Mycology Research Center, Chiba UniversityChiba, Japan; ^3^Faculty of Pharmaceutical Sciences, Health Sciences University of HokkaidoHokkaido, Japan; ^4^Kazusa DNA Research InstituteChiba, Japan

**Keywords:** *Panax japonicas*, RNA-seq, triterpenoid saponins, cytochrome P450, comparative transcriptomics analysis

## Abstract

The *Panax* genus has been a source of natural medicine, benefitting human health over the ages, among which the *Panax japonicus* represents an important species. Our understanding of several key pathways and enzymes involved in the biosynthesis of ginsenosides, a pharmacologically active class of metabolites and a major chemical constituents of the rhizome extracts from the *Panax* species, are limited. Limited genomic information, and lack of studies on comparative transcriptomics across the *Panax* species have restricted our understanding of the biosynthetic mechanisms of these and many other important classes of phytochemicals. Herein, we describe Illumina based RNA sequencing analysis to characterize the transcriptome and expression profiles of genes expressed in the five tissues of *P. japonicus*, and its comparison with other *Panax* species. RNA sequencing and *de novo* transcriptome assembly for *P. japonicus* resulted in a total of 135,235 unigenes with 78,794 (58.24%) unigenes being annotated using NCBI-nr database. Transcriptome profiling, and gene ontology enrichment analysis for five tissues of *P. japonicus* showed that although overall processes were evenly conserved across all tissues. However, each tissue was characterized by several unique unigenes with the leaves showing the most unique unigenes among the tissues studied. A comparative analysis of the *P. japonicus* transcriptome assembly with publically available transcripts from other *Panax* species, namely, *P. ginseng, P. notoginseng*, and *P. quinquefolius* also displayed high sequence similarity across all *Panax* species, with *P. japonicus* showing highest similarity with *P. ginseng*. Annotation of *P. japonicus* transcriptome resulted in the identification of putative genes encoding all enzymes from the triterpene backbone biosynthetic pathways, and identified 24 and 48 unigenes annotated as cytochrome P450 (CYP) and glycosyltransferases (GT), respectively. These CYPs and GTs annotated unigenes were conserved across all *Panax* species and co-expressed with other the transcripts involved in the triterpenoid backbone biosynthesis pathways. Unigenes identified in this study represent strong candidates for being involved in the triterpenoid saponins biosynthesis, and can serve as a basis for future validation studies.

## Introduction

Medicinal plants are a rich source of diverse bioactive compounds that play an important role in the management of human health. Availability and understanding of the phytogenome sequences, and transcriptomics information of medicinal plants provide a broad understanding of different ongoing metabolic processes, their localization across different plant tissues, and the cellular biosynthetic mechanisms involved in their biosynthesis (Rai and Saito, 2015). Complete genome sequencing of a medicinal plant has its own challenges which include the complexity of the genome and the sequencing cost, and the computational resources required, resulting in only few complete genomes of medicinal plants that are available. Recent advancements in the next generation sequencing (NGS) with reduced operational cost for such experiments, and development of computational resources to perform *de novo* transcriptome assembly, annotation, and characterization have revolutionized the field of natural medicine (Muranaka and Saito, 2013; Saito, 2013). The information from transcript sequences and their relative abundance across tissues in a medicinal plant provide key insight on important aspects of gene structure, function, and regulation including non-coding RNAs or differentially expressed genes.

Among medicinal plants, *Panax japonicus* CA Meyer, along with other *Panax* species such as *P. notoginseng, P. quinquefolius*, and particularly, *P. ginseng* are the most popular and among the list of the best-selling medicinal plants in the world (Briskin, 2000; Qi et al., 2011). ‘Panax’ is derived from a Greek word, ‘Panacea,’ which means cure for all. It includes 12 established species, *P. japonicus* being one of them, with useful medicinal properties (Han et al., 2013). *P. japonicus*, also known as ‘Chikusetsu-ninjin’ in Japan, belongs to the genus *Panax* of the family *Araliaceae*, and grows wild throughout Japan, China and Korea with a characteristic long bamboo-like rhizome (Morita et al., 1985). It has been used as a traditional medicinal herb and as a substitute to *P. ginseng* in minority ethnic group for 1000 years (Yang X. et al., 2014).

The rhizome extracts from *P. japonicus* have reportedly been used for the treatment of several life-style related diseases, such as arteriosclerosis, hyperlipidaemia, hypertension, and non-insulin dependent diabetes (Vogler et al., 1999; Han et al., 2005). Extracts from dried rhizome of *P. japonicus* have been used for 100s of years in Japan as a drug for gastroenteric disorder, anti-ulcer, expectorants, and anti-pyretic, while in China, it have been used as tonic, anti-inflammatory, and haemostatic agents (Zou et al., 2002). According to the description in the “Ben Cao Gong Mu Shi Yi,” rhizomes of *P. japonicus* possess combined medicinal properties of “conserving vitality” and “replenishing blood” activities attributed to *P. ginseng* and *P. notoginseng*, respectively, and therefore was named as the “king of herbs” in the traditional Tujia and Hmong medicine (Zhang et al., 2015). *P. japonicus* has been included in the Chinese and Japanese pharmacopeia for its various pharmacological properties, such as anti-ulcer, anti-obesity, analgesic, anti-fatigue, anti-oxidant, anti-cancer, and immuno-regulation (Yamahara et al., 1987; Yun, 2001; Han et al., 2005; Yang X. et al., 2014). Despite of well-documented medicinal properties of *P. japonicus*, little is known about its transcriptome, and its genome is not sequenced.

For *Panax* species, most of their therapeutic effects have been attributed to its metabolic extracts being rich in pharmacologically active constituents, known as ginsenosides (Shibata, 2001; Murthy et al., 2014; Yang W.Z. et al., 2014). Ginsenosides are the oligosaccharide glycosides of dammarane- or oleanane-type triterpenoids. Ginsenosides biosynthetic pathway derives its precursor from the triterpene backbone pathways, starting from acetyl-CoA toward the synthesis of 2,3-oxidosqualene, cyclization of which forms the branch point between dammarane- and oleanane-type terpenoids. These reactions are catalyzed by the cytochrome P450 enzymes, little of which involved in ginsenosides biosynthesis are known in *Panax* species (Han et al., 2011, 2013). Triterpenoids are synthesized through one of the major isoprenoid biosynthetic pathway, and are one of the most important classes of natural product, glycoside conjugation of which has important medicinal properties. Ginsenosides saponins are highly diversified, with 289 different types of saponins being isolated and characterized so far in pure form from either eleven different *Panax* species, or chemically derived from their extracts (Yang W.Z. et al., 2014). Ginsenosides composition varies significantly across *Panax* species, with *P. ginseng, P. notoginseng, P. quinquefolius*, and *P. vietnamisis* being grouped in the dammarane-type saponins rich species, while *P. japonicus, P. zingiberensis*, and *P. stipuleanatus* being grouped in the oleanane-type saponins rich species (Zhu et al., 2004). The total saponins content in *P. japonicus* rhizome can reach to 15% of the total content, which was 2- to 7-fold higher than that of *P. ginseng*, and threefold higher than that of *P. quinquefolius* (Yun, 2001). *P. japonicus* saponins content were found to be rich in oleanane-type saponins, with the ratio of dammarane- to oleanane-type saponins being less than 0.25 (Zhu et al., 2004). Several oleanane-type saponins, such as chikusetsusaponins-1b, -IV, -IVa, and -V, along with dammarane-type saponins, such as chikusetsusaponins-1a, and -111 among others have been isolated and characterized from *P. japonicus* (Morita et al., 1985; Zou et al., 2002; Yoshizaki et al., 2013).

Despite pharmacological importance of *P. japonicus*, the transcriptome and genome data are limited, with only 867 sequences being available in the NCBI database, out of which, 788 sequences are associated with chloroplast. The limited transcriptome data hinders identification of key factors that regulate saponins biosynthesis in *P. japonicus*. Further, genes encoding cytochrome P450 (CYP450s) and glycosyltransferases (GTs), and involved in the ginsenosides biosynthesis are not known. As ginsenosides are synthesized in all of the *Panax* species, transcriptome comparison across *Panax* genus will be useful to identify potential candidates for future validation.

In this study, we have established *de novo* transcriptome assembly for *P. japonicus* using five of its tissues, namely, flower, leaf, secRoot (secondary root), rhizome_Y (young rhizome tissue), and rhizome_O (old rhizome tissue). We identified putative genes from *P. japonicus* involved in the triterpene backbone biosynthetic pathways. Moreover, the comparative transcriptome analysis of *P. japonicus* with three major *Panax* species allowed us to identify potential candidate genes which may be involved in the synthesis of ginsenosides. This study, therefore, may serve as a basis for the future discoveries on functional genes involved in the ginsenosides biosynthesis and regulation.

## Materials and Methods

### Plant Materials

All five tissues, namely flower, leaf, secRoot, rhizome_Y, and rhizome_O were harvested in the month of June, 2014 from a 7-years-old *P. japonicus* plant, growing in the natural environment at the medicinal plant garden, Health Sciences University of Hokkaido, Japan. A voucher specimen (accession no. E4532-42-0003-3-C4) was deposited in the Herbarium of Faculty of Pharmaceutical Sciences, Health Sciences University of Hokkaido. For rhizome, we collected first bud scars, nearest to the stem, and called as rhizome_Y, and sixth bud from the stem, and called as rhizome_O. All tissues were cut into small pieces, frozen by liquid nitrogen, and stored at -80°C prior to RNA extraction.

### RNA Isolation, and cDNA Synthesis

The frozen samples of *P. japonicus* were powdered using a Multi Beads Shocker (Yasui Kikai, Japan), and used for subsequent RNA extraction. Total RNA was extracted using the RNeasy Plant Mini Kit (Qiagen, USA), according to manufacturer’s instruction. The extracted RNA was treated with RNase-free DNase (TaKaRa, Japan), purified on a RNA-purification column (Qiagen, USA), and finally collected by ethanol precipitation. The RNA quality was evaluated on the Agilent Bioanalyzer 2100 (Agilent Technologies, USA), and RNA samples with RIN (RNA Integrity Number) value above 8 was used for subsequent cDNA synthesis.

To create cDNA sequencing library, mRNAs with poly(A) tail were isolated from the total RNA using beads with oligo(dT). Fragmentation buffer was then added to shear mRNAs into short fragments, which then served as templates for the synthesis of first strand of cDNA using random hexamer primers. cDNA library was then prepared using SureSelect Strand Specific RNA Library kit (Agilent Technologies, USA) according to the manufacturer’s specifications.

### Illumina Sequencing

Paired-end reads with an average length of 101 bps were generated by sequencing cDNA libraries on an Illumina HiSeq^TM^ 2000 sequencer (Illumina, Inc., USA). Library construction and sequencing were performed at Kazusa DNA Research Institute, Chiba, Japan. After the removal of adapter sequences, empty reads, reads with ambiguous ‘N’ bases > 5%, raw reads of low quality (Q < 20), and raw reads with an average length less than 50 bases, Illumina sequencing resulted in a total of over 24 million paired end clean reads for *P. japonicus*. The raw read sequences for all five tissues of *P. japonicus* discussed in this publication have been deposited in NCBI’s Gene Expression Omnibus (GEO; Edgar et al., 2002), and are accessible through GEO Series accession number GSE78893^1^.

### Data Pre-processing, and *De novo* Transcriptome Assembly

Filtering of raw reads were performed using Trimmomatic program (Bolger et al., 2014), resulting in paired end clean reads, and unpaired reads which lost its corresponding sequence partner due to quality control. Tissue-wise distribution of paired read counts, and resulting clean paired reads after pre-processing through Trimmomatic program is shown in the **Supplementary Table S1**. The Trinity program v2.0.6 was used for the *de novo* transcriptome assembly using clean reads from all the samples. We then processed these contigs for read alignment and abundance estimation using Bowtie 2.0 (Langmead et al., 2009), and RSEM (Li and Dewey, 2011), respectively. To calculate unigene expression, we used Fragments per Kilobase exon per Million mapped fragments (FPKM) method. GC content and basic statistics values for contigs and unigenes were calculated as described previously (Fukushima et al., 2015).

### Functional Annotation and Classification of Unigenes

We performed BLASTx program based homology search for the *P. japonicus de novo* transcriptome assembly against the NCBI-nr protein database (^2^formatted on April 30, 2015) using a cutoff E-value < 10^-5^, and maximum number of allowed hit fixed at 20. The alignment results with the smallest E-value were used to annotate all the unigenes. For their further annotation and classification, we used the Blast2GO program v 3.0 to assign GO terms, an EC number, and KEGG information to the unigenes using associated BLASTx search results. Blast2GO v 3.0 (Biobam, Spain) was used for the visualization of the GO functional classification of all the unigenes and the distribution of the gene functions in different species. To perform k-mean clustering, we used Genespring 13.0 (Agilent Technologies, USA), with distance matrix being calculated by Euclidean similarity measurement with 10,000 iterations and number of clusters being set to six.

### Simple Sequence Repeat (SSR) Detection

All *P. japonicus* transcripts were searched to determine the composition, frequency, and distribution of simple sequence repeats (SSRs) by using the microsatellite identification tool (MISA; Thiel et al., 2003^3^). The search parameters for maximum motif length group were set to recognize hexamers with each SSRs length based category to have at least five repeats.

### GO Enrichment Analysis

Unigenes with the FPKM value over 10 in each of the tissues were used as a test set, and compared against the whole transcriptome background of *P. japonicus* for GO enrichment analysis. GO enrichment analysis was performed on all of the tissues using Blast2GO v3.0 based on Fisher’s exact test, and a *P*-value cutoff of 0.05 (hypergeometric test with Benjamini and Hochberg false discovery rate correction) was applied.

### Phylogenetic Analysis

Using Biology Workbench online tool^4^, we translated selected unigenes sequences from *P. japonicus*, and obtained corresponding protein sequences by selecting the translation frame that started with methionine and had longest amino acid sequences. Protein sequences from unigenes were combined together with protein sequences from the databases, aligned using MUSCLE program, and evolutionary distances were computed using Jones-Taylor-Thornton (JTT) method, and a Neighbor-Joining (NJ) tree was constructed with bootstrap values obtained after 1000 replications using MEGA6 software (Tamura et al., 2013).

## Results

### Sample Preparation and Illumina Sequencing

**FIGURE 1:**
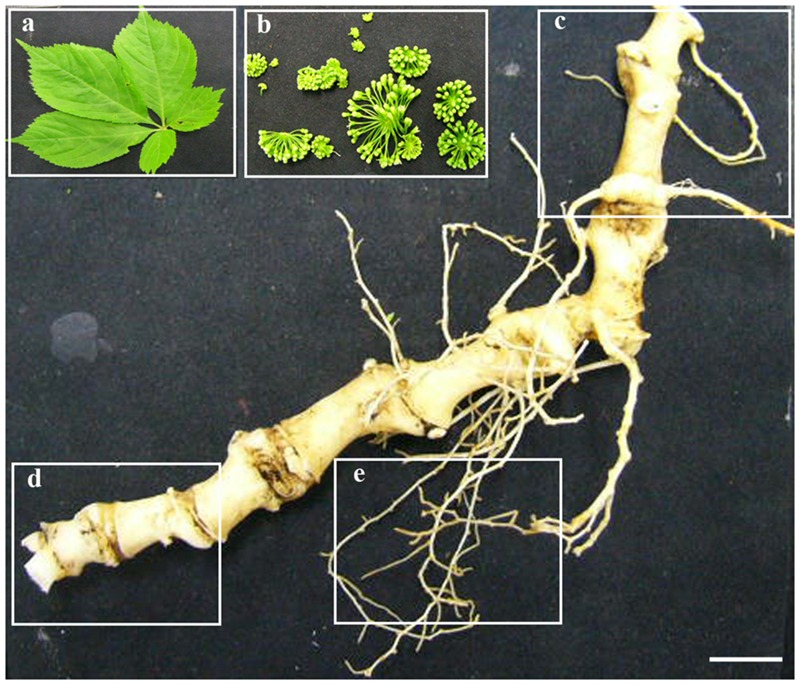
**Five tissues of *P. japonicus* used for transcriptome study.** Five tissues of *P. japonicus*, namely, leaf **(a)**, flower **(b)**, rhizome_Y **(c)**, rhizome_O **(d)**, and secRoot **(e)** were used to performed transcriptome profiling, and *de novo* transcriptome assembly. Scale bars for each panel measure to 0.5 cm.

*Panax japonicus* plant is characterized by 3–5 leaflets, with a characteristic bamboo-like rhizome. As the stem (‘sympodium’) dies back each fall, it leaves a bud scar on the root collar or rhizome, which then are used as a means of assessing age of the plant. To study the transcriptomes of *P. japonicus*, five tissues, namely flower, leaf, secRoot (secondary root), rhizome_Y (young rhizome, first bud scar below the stem), and rhizome_O (old rhizome, sixth and seventh bud scar below the stem) were collected from 7-years-old plant (**Figure 1**). Total RNA for each sample were selected for the mRNA preparation, fragmentation, cDNA synthesis, and library preparation. Each library, thus prepared, was sequenced using the Illumina HiSeq^TM^ 2000 platform. Adapter sequences, low-quality reads, and reads that were shorter than 50 base pairs (bps) were removed, yielding to a total of over 24 million 100 bps paired end clean reads, or approximately 2.4 Gbps in total. Mean phred score, a bench mark to access the quality of the short reads, across all tissues were above 36.5, indicating that our RNA sequencing was adequate for the *de novo* transcriptome assembly. The study overview is shown in the **Supplementary Figure S1**.

### *De novo* Transcriptome Assembly and Gene Expression Profiling

**FIGURE 2:**
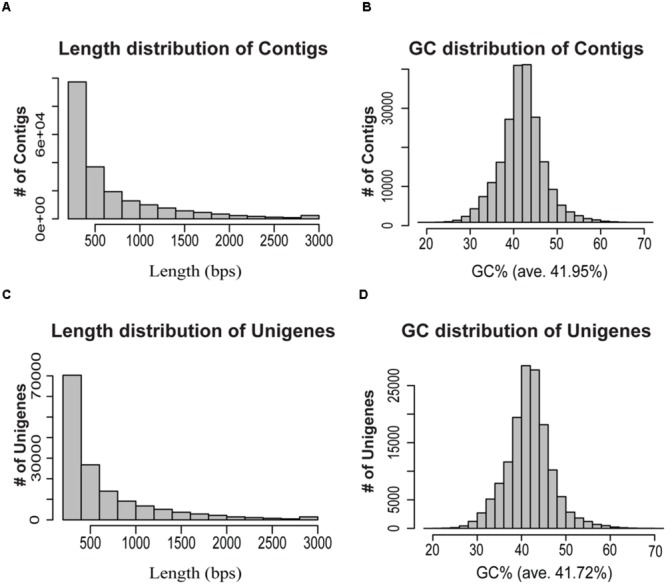
**Overview of the *de novo* transcriptome assembly in *P. japonicus*. (A,B)** represents length and GC distribution of contigs assembled from all cleaned reads from five tissues of *P. japonicus* using Trinity program (Grabherr et al., 2011), **(C,D)** represents length and GC distribution of unigenes generated from further contigs assembly.

Clean reads from all samples were pooled together, and were further assembled *de novo* using Trinity program (Grabherr et al., 2011), resulting in 206,610 contigs (138,140,893 bps) with an average length of 668 bps and an N50-value of 950 bps (**Table 1**). The length and GC% distribution of all contigs have been shown in **Figures 2A,B**. We obtained 135,235 unigenes (77,297,613 bps) with an average length of 571 bps, and an N50-value of 889 bps, with length and GC% distribution as shown in **Figures 2C,D**. The length of assembled unigenes ranged from 200 to 11,387 bps, with 70,309 unigenes being shorter than 500 bps, and 1,258 unigenes sequence lengths being more than 3,000 bps. To estimate the expression abundance, clean reads were mapped to the *P. japonicus de novo* transcriptome assembly using Bowtie 2.0 (Langmead et al., 2009), and expression abundance was calculated using RSEM program (Li and Dewey, 2011).

**Table 1:** Summary of the sequence assembly after Illumina sequencing.

	Contiqs	Unigenes
Number of contigs	206,610	135,235
Average length (bps)	668.61	571.58
Median length (bps)	423	356
Max length (bps)	11,387	11,387
Min length (bps)	200	200
N50 (bps)	957	889
Nn50	42,525	30,644
Total assembled bases	138,140,893	77,297,613

### Functional Annotation and Classification of *P. japonicus* Unigenes

**FIGURE 3:**
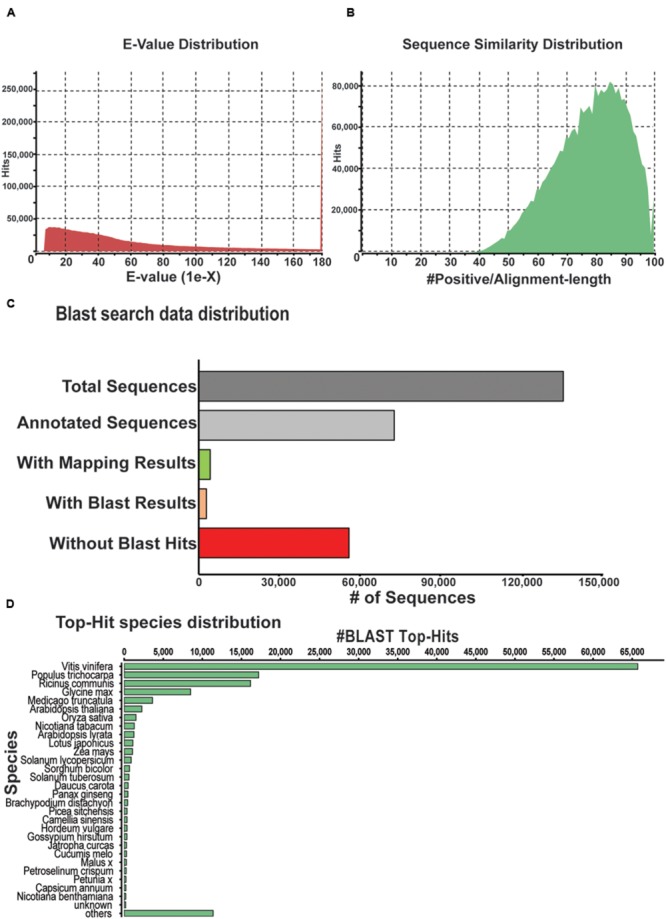
**Characterization of *P. japonicus* unigenes based on NCBI non-redundant (nr) protein database search. (A)** E-value distribution of Blast hits for the *P. japonicus* assembled transcriptome with a cutoff of E-value < 10^-5^. **(B)** Similarity score distribution plot of top Blast hits for the assembled unigenes. **(C)** Bar chart of the data distribution based on blast search and annotation using Blast2GO. **(D)** Species distribution of the top Blast hits for the assembled unigenes.

The unigenes derived from the *P. japonicus* transcriptome assembly were subjected to additional validation and annotation. BLASTx program (Altschul et al., 1997) based homology search was conducted against an NCBI non-redundant (nr) protein database for all unigenes, and best aligning results were selected to annotate the unigenes. This resulted in 87.2% of aligned sequences displaying significant homology with the NCBI-nr database (**Figure 3A**). Based on the BLAST similarity distribution, over 80,000 sequences of *P. japonicus* exhibited alignment identity greater than 85% (**Figure 3B**). Blast2GO program v 3.0 (Conesa and Gotz, 2008) was then used to obtain gene ontology (GO), to assign an Enzyme Commission (EC) number, and Kyoto Encyclopedia of Gene and Genomes (KEGG; Kanehisa et al., 2014) based annotation to the unigenes based on the BLAST results. A total of 78,794 unigene (58.26%) were annotated with a minimum of one biological term from GO or KEGG pathway information (**Figure 3C**), while remaining 56,441 unigenes resulted in no BLAST hit. These unannotated unigenes may yet be uncharacterized genes or assembled sequences that were too small to produce hits in the search.

Top-hit species distribution analysis based on BLASTx results showed 75% of unigenes with blast hit sharing high sequence similarity with sequences of *Vitis vinifera* (47.6%), *Populus trichocarpa* (12.47%), *Ricinus communis* (11.71%), and *Glycine max* (6.14%; **Figure 3D**). Among other top-hit species, *P. ginseng* was placed at 16th position, with 470 unigenes from *P. japonicus* showing top sequences similarity. High sequence similarity of *P. japonicus* with that of *P. ginseng* was anticipated, and is consistent with earlier studies that suggests *P. japonicus* and *P. ginseng* to be closest among the rest of the *Panax* species (Zhu et al., 2003; Choi et al., 2011).

**FIGURE 4:**
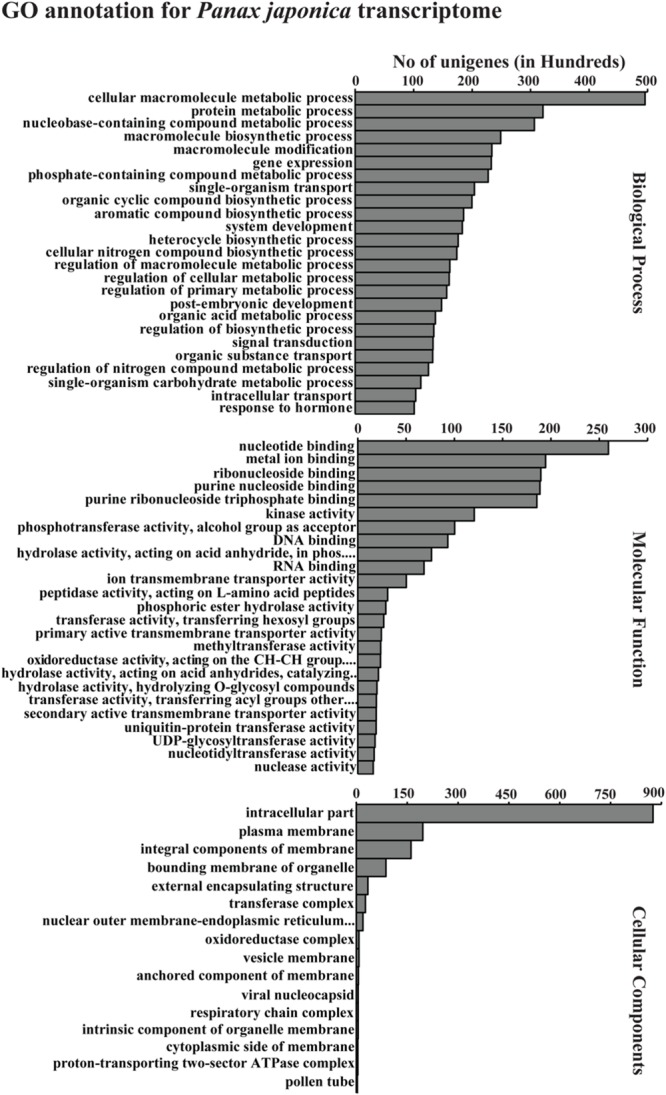
**Gene ontology (GO) annotation for all assembled unigenes in *P. japonicus*.** GO-terms for all unigenes were assigned based on Blast search results using Blast2GO program at GO annotation level 5. The results are summarized in terms of three functional categories, namely, biological process, molecular function, and cellular component.

Gene ontology functional classification for the *P. japonicus* transcriptome was performed using Blast2GO at the annotation level 5, resulting in 66 GO categories; molecular function (25), biological process (25), and cellular component (16; **Figure 4**). Among molecular function category, most noticeably, GO terms corresponding to different hydrolase activities, hydrolase activity that involves hydrolysing *o*-glycosyl compounds, transferase activity that involves transferring hexosyl groups, and UDP-glycosyltransferase activity were abundant. Within the biological process category, metabolic processes, such as the cellular macromolecular metabolic process, protein metabolic process, nucleotide-containing compound metabolic process, macromolecule biosynthetic process, aromatic compound biosynthetic process, and organic acid metabolic process were among most abundant within biological processes category. Within cellular component category, GO terms corresponding to the intracellular part, plasma membrane, integral components of membrane, bounding membrane of organelle, external encapsulating structure and transferase complex were abundant among unigenes.

### Functional Classification of KEGG Pathways

The use of the KEGG pathway database facilitates the functional annotation of cellular components and their interactions within various biological pathways. This ”pathway” based annotation provides an overview of the different active metabolic processes within an organism, and therefore, enables further understanding of the biological functions of the unigenes. Using Blast2GO, all unigenes were mapped against the KEGG database, resulting in 17,394 unigenes grouped into 146 KEGG pathways (**Supplementary Table S2**). Purine metabolism, thiamine metabolism, starch and sucrose metabolism, aminobenzoate degradation, glycolysis, and phenylpropanoid biosynthesis pathways were the most represented pathways observed. Terpenoid backbone biosynthesis (map00900), a key pathway for the synthesis of saponins, was assigned to 322 of these unigenes.

### Identification of Simple Sequence Repeats (SSRs)

Simple sequence repeats are ubiquitously distributed tandem iterations of short oligonucleotides within a genome, consisting of simple motifs of 1–6 nucleotides that are repeated from two to a few dozen times at a locus. SSRs being an indel mutational hotspots in genomes are important markers for determining genetic variations including paternity determination, genetic diversity assessment, population genetics studies, and for the development of genetic map (Hancock, 1996; Varshney et al., 2005). There have been several reports that implicate SSRs in affecting genes expression, and that polymorphism of SSRs tracts may be important in the evolution of gene regulation (Varshney et al., 2002; Jarvis et al., 2008; Senthilvel et al., 2008). To identify SSRs for *P. japonicus*, all the contigs for mono- to hexa-nucleotide motifs were searched, with a minimum of five repetitions using the software MISA (Thiel et al., 2003). We identified a total of 30,966 SSRs present across 26,557 contigs of *P. japonicus*, with 3,742 contigs showed presence of more than one SSRs (**Table 2**). Among the different SSRs repeat-type classes, the mono-nucleotide represented the largest fraction (48.64%) of identified SSRs, followed by di-nucleotide (23.94%) and tri-nucleotide (23.37%). Numbers of SSRs for tetra-, penta-, and hexa-nucleotide repeat classes were significant but relatively small compared to the mono-, di-, and tri-SSR repeats. All detected SSRs for *P. japonicus* are shown in **Supplementary Table S3**. Herein identified SSRs of *P. japonicus* may provide a potential genetic marker, which will be useful for performing population genetics and comparative genomics studies across the *Panax* species synthesizing saponins.

**Table 2:** Statistics of SSR detected in *Panax japonicus.*

Results of SSR searches	
Total number of sequences examined	206,610
Total size of examined sequences (bps)	138,140,893
Total number of identified SSRs	30,966
Number of SSR containing sequences	26,557
Number of sequences containing more than 1 SSR	3,742
Number of SSRs present in compound formation	1,662

**Distribution to different repeat type classes**	

Di-nucleotides	15,064
Tri-nucleotides	7,416
Tetra-nucleotides	7,237
Penta-nucleotides	6,24
Hexa-nucleotides	3,81

### Functional Classification and Comparison of Highly Expressed Transcripts across Five Tissues of *P. japonicus*

**FIGURE 5:**
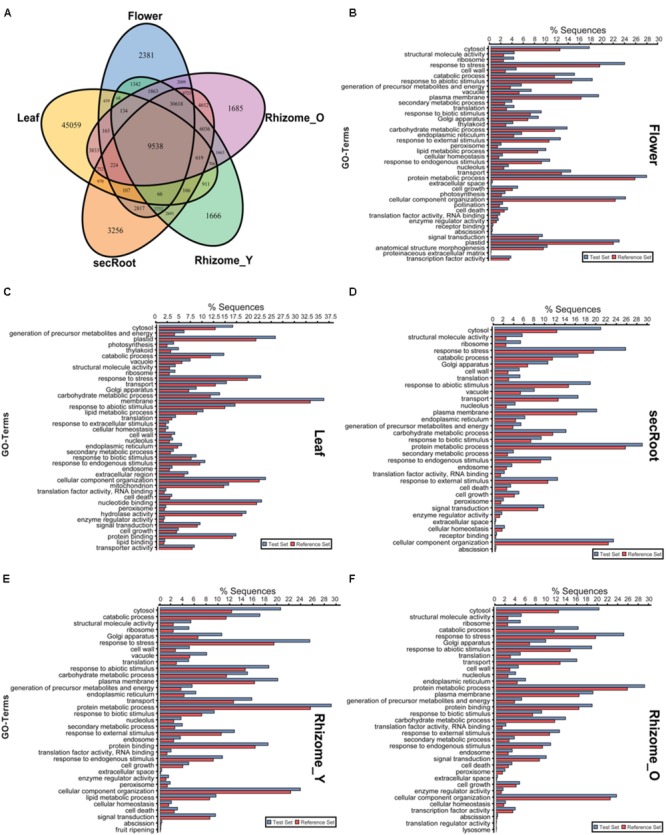
**Distribution of unigenes and gene ontology enrichment analysis for five tissues of *P. japonicus*. (A)** Venn diagram for unigenes with non-zero FPKM values in individual tissues. **(B–F)** Gene ontology enrichment analysis using Fisher’s exact test with *P*-value cutoff applied as 0.05. Unigenes for each tissue with FPKM value above 10 were selected as test set, and used against the *P. japonicus* transcriptome assembly as reference set to identify differential GO-terms enriched in the specific tissue, with *P*-value being calculated based on reference set.

A comparative analysis of expression of the unigenes (presence or absence) across and within all the five tissues of *P. japonicus* showed 59,228, 63,757, 69,969, 60,819, and 69,222 unigenes with non-zero FPKM values for flower, leaf, secRoot, rhizome_Y, and rhizome_O, respectively. While numbers of unigenes with non-zero expression value were comparable across all five tissues, each showed several unique unigenes specific to that tissue. Among all unigenes, 9,534 unigenes were shared across all tissues (**Figure 5A**), while 2,381, 45,059, 3,256, 1,666, and 1,685 unigenes were specifically found in the flower, leaf, secRoot, rhizome_Y and rhizome_O, respectively. With the exception of leaf, other tissues of *P. japonicus* showed overlap of unigenes with non-zero expression values across all tissues. Overall, our results showed that the transcriptome profile for rhizome_Y and rhizome_O tissue were highly similar with sharing 48,808 unigenes, and were significantly similar to secRoot (**Supplementary Figure S2**).

To compare the distribution of differentially enriched GO-terms across five tissues of *P. japonicus* analyzed for the highly expressed transcripts, we performed GO-term enrichment analysis using Fisher’s exact test by selecting unigenes for each tissue with FPKM value greater than 10, and were further tested against *P. japonicus* transcriptome assembly (**Figures 5B–F**). Flower’s unigenes showed 40 GO-terms being enriched, with GO-terms corresponding to pollination, anatomical structure morphogenesis and proteinaceous extracellular matrix were specific to flower. GO-term enrichment analysis for the leaf resulted into 40 GO-terms, 8 of which were specific to leaf, where the top four enriched GO-terms for leaf were cytosol, generation of precursor metabolites and energy, plastid and photosynthesis. Enriched GO-terms for secRoot, rhizome_Y and rhizome_O were highly similar, with only one GO-term corresponding to fruit ripening was specific to rhizome_Y.

### Comparison of Assembled Unigenes with *P. ginseng, P. notoginseng*, and *P. quinquefolius* Published Transcriptome

A comparative examination of the transcriptome assembly of *P. japonicus* with three *Panax* species, namely, *P. ginseng, P. quinquefolius*, and *P. notoginseng* was undertaken. Searching for *Panax* related nucleotides and ESTs from NCBI resulted in a total of 106,320 sequences being deposited for *P. ginseng*, while for the other *Panax* species, the number of sequences were limited. For *P. ginseng*, we obtained all nucleotide sequences deposited in NCBI and used for comparison with *P. japonicus*. For comparison with *P. quinquefolius*, we obtained its annotated transcriptome assembly (pqa_assembly_v_10072011) from medicinal plant genomics resource consortium^5^. As number of transcripts for *P. notoginseng* in the NCBI database were less than 300, we obtained raw sequence datasets from NCBI-SRA (sequence read archive) database as described in the material and methods, with specific experiments used in this study are listed in the **Supplementary Table S4**. Using this dataset, we performed *de novo* transcriptome assembly for *P. notoginseng* transcriptome with the Trinity software that resulted in 186,450 unigenes with N50-value 735 bps (**Supplementary Table S4**).

We performed two-way blastn search to compare *P. japonicus* with other *Panax* species (**Supplementary Figure S1**). In the first case, we used *P. japonicus* transcriptome as query against sequences from individual *Panax* species transcriptome selected for this study as blastn database, resulting in 51.56, 63.14, and 53.13% of its contigs having a hit against *P. ginseng, P. notoginseng*, and *P. quinquefolius*, respectively (**Table 3**). Overall, a total of 1,34,387 transcripts of *P. japonicus* got hit against at least one of the *Panax* species, of which, 90,951 contigs (67.67%) showed sequence similarity against corresponding sequences in all *Panax* species, while 72,223 contigs were unique for *P. japonicus* with no hit to any *Panax* species (**Supplementary Figure S3**).

**Table 3:** Comparative transcriptomic analysis of *P. japonicus* with other three *Panax* species based on Blastn sequence similarity.

Query	No of query genes	No of query genes with blast hit	No of query genes with no blast hit	Percentage of genes with blast hit	Databases
*Panax ginseng*	106,320	95,218	11,102	89.55	*Panax japonicus*
*Panax notoginseng*	254,971	156,801	98,170	61.49	*Panax japonicus*
*Panax quinquefolius*	110,795	92,497	18,297	83.48	*Panax japonicus*
*Panax japonicus*	206,610	106,530	100,080	51.56	*Panax ginseng*
*Panax japonicus*	206,610	130,462	76,148	63.14	*Panax notoginseng*
*Panax japonicus*	206,610	109,782	96,828	53.13	*Panax quinquefolius*

We next performed blastn search using each of the individual *Panax* species transcriptome as a query against the *P. japonicus* transcriptome as a blastn database. Among *Panax* species, 89.55, 61.49, and 83.48% of the sequences used as a query from *P. ginseng, P. notoginseng*, and *P. quinquefolius*, respectively, showed sequence similarity with at least one transcript of *P. japonicus* (**Table 3**). *P. ginseng*, and *P. quinquefolius* showed higher sequence similarity with *P. japonicus*, with majority of its sequences matched with the corresponding sequences in the *P. japonicus*, while *P. notoginseng* showed relatively less similarity with *P. japonicus*. Previous studies using SSR markers for the assessment of genetic diversity in the *Panax ginseng* cultivars and related species concluded that *P. japonicus* and *P. ginseng* are phylogenetically similar, while *P. notoginseng* and *P. quinquefolius* are relatively distant with *P. japonicus* (Choi et al., 2011). While high sequence similarity does not mean phylogenetic closeness, but if two species are phylogenetically closer, then we would expect higher sequence similarity compared to other species. Therefore, our results were consistent with previous reports, showing *P. japonicus* and *P. ginseng* sharing high sequence similarity.

### Identification of Unigenes Putatively Involved in Triterpene Saponins Biosynthesis

**FIGURE 6:**
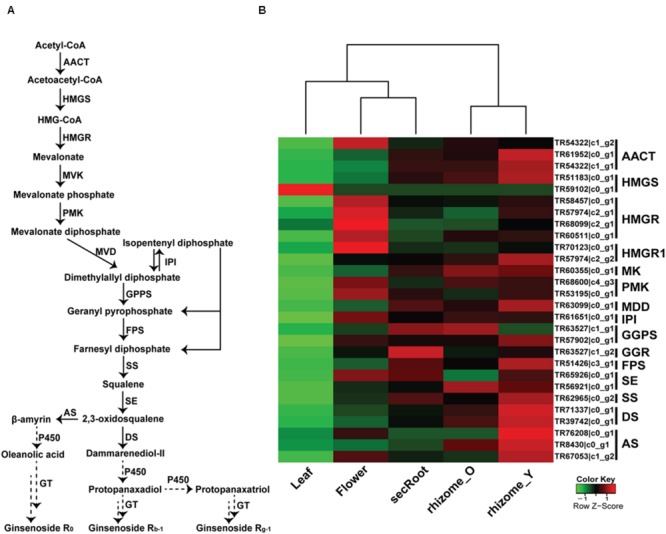
**Putative triterpenoid saponins biosynthesis pathway and expression level of associated unigenes across five tissues in *P. japonicus*. (A)** Proposed pathway for triterpenoid saponins biosynthesis. **(B)** Expression levels of the candidate unigenes that showed high sequence similarity with other *Panax* species, and were annotated as enzyme coding genes from triterpenoid backbone biosynthesis pathways. The expression value (FPKM) for unigenes across all tissues were log_2_ transformed and scaled across each row, and heatmap was generated using R-package heatmap2.0. AACT, Acetyl-CoA acetyltransferase; HMGS, hydroxymethylglutaryl-CoA synthase; HMGR, hydroxymethylglutaryl-CoA reductase; MVK, mevalonate kinase; PMK, phosphor mevalonate kinase; MVD, mevalonate diphosphate decarboxylase; IPI, isopentenyl diphosphate isomerase; GPPS, geranylgeranyl pyrophosphate synthase; FPS, farnesyl diphosphate synthase; SS, squalene synthase; SE, squalene epoxidase; DS, dammarendiol synthase; AS, beta-amyrin synthase; GTs, UDP glycosyltransferase; P450, cytochrome P450; FPKM, fragments per kilobase per million.

Triterpene saponins are derived from terpenoid backbone biosynthesis, followed by sesquiterpenoid and triterpenoid biosynthesis, which are then used as precursors by specific CYP450s and GTs for the formation of various ginsenosides (**Figure 6A**). Using annotated *P. japonicus* transcriptome assembly and blastn results of *P. japonicus transcripts* against other *Panax* species, we identified 28 unigenes with sequence length more than 500 bps, annotated as known enzymes involved in the mevalonate (MVA) pathway for triterpene saponins biosynthesis, and showing above 90% sequences similarity against all *Panax* species (**Figure 6B**). A heat-map, representing gene expression of these unigenes, showed differential expression across the various *P. japonicus* tissues, with genes involved in the triterpene saponins biosynthesis displaying higher expression in the rhizome_Y tissue, followed by rhizome_O, and secRoot. A hierarchical clustering of unigenes for triterpene saponins biosynthesis pathways showed three distinct groups, with the rhizome_Y and rhizome_O being in one group, flower and secRoot in the second group, and the leaf being the third group, where the later showed the least expression of all selected unigenes.

### Identification of Candidate Unigenes Putatively Involved in Ginsenosides Biosynthesis

**FIGURE 7:**
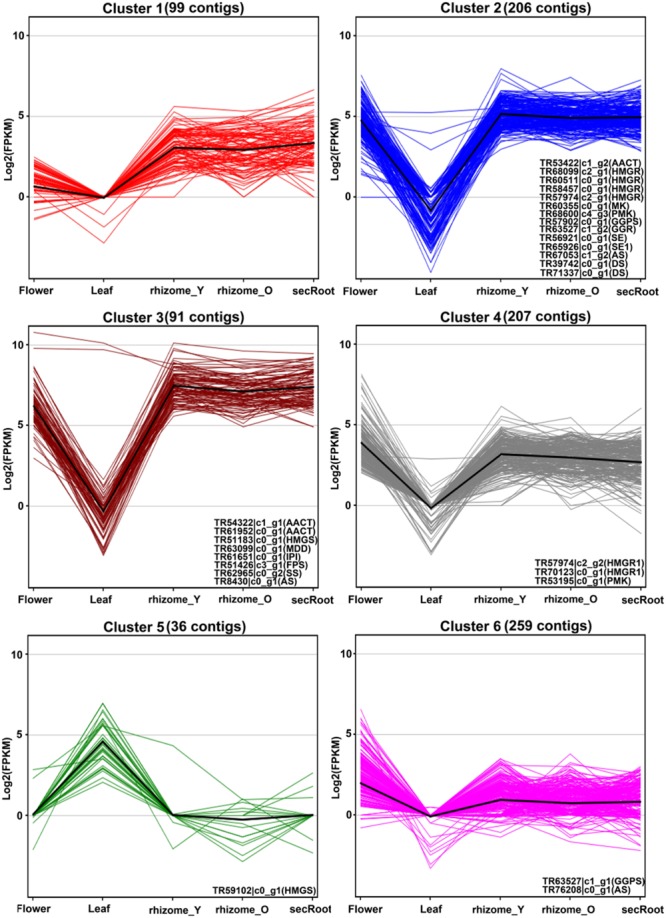
**K-mean clustering for unigenes annotated as CYP450s or associated with sugar conjugation processes together with unigenes from triterpenoid backbone biosynthetic pathways.** Unigenes annotated as cytochrome P450, or sugar conjugation associated processes were selected from the *P. japonicus* transcriptome assembly, resulting in total of 870 unigenes. These gene groups were used together with 28 unigenes annotated as enzyme coding genes from triterpenoid backbone biosynthetic pathways, and k-mean clustering analysis was performed based on gene expression values across all five tissues of *P. japonicus*. Distance matrix for k-mean clustering was calculated by Euclidean similarity measurement with 10,000 iterations, resulting in 6 gene clusters. Name of unigenes associated with triterpenoid backbone biosynthetic pathways and identified in a particular cluster is represented within each gene group.

It has been suggested that putative genes involved in the triterpene saponins biosynthesis are mainly CYP450s and GTs enzyme coding genes, which may account for the synthesis and accumulation of triterpene saponins in specific tissues. In order to identify these putative genes associated with ginsenosides biosynthesis, we selected 870 *P. japonicus* unigenes annotated as CYP450s or associated with sugar conjugation, and have sequence similarity with all *Panax* species. These, together with the 28 unigenes associated with MVA pathway for triterpene saponins biosynthesis, were used to perform k-mean clustering using Euclidean similarity measurement with 10,000 iterations. This resulted in all unigenes being grouped in six different clusters (**Figure 7** and **Supplementary Table S5**). Among gene clusters, cluster-2 and -3 (297 unigenes) included 22 out of 28 genes corresponding to triterpene saponins biosynthesis. Cluster-2 and -3 were unique with respect to other gene clusters, as expression of all unigenes were highest in the rhizome_Y and rhizome_O, while lowest in the leaf. Within cluster-2 and -3, the median expression of all unigenes in the rhizome_Y was 5.13 and 7.39, respectively. Although, the expression trend for cluster-4 was similar to cluster-2 and -3, and included three unigenes associated with MVA pathways, the median expression values for the unigenes were low. Inspection of the unigenes annotated as CYP450s within cluster-4 revealed a unigene TR49263_c0_g1 annotated as CYP716A53v2 (JX036031), a gene characterized as protopanaxadiol 6-hydroxylase producing protopanaxatriol triterpene aglycone from protopanaxadiol in *P. ginseng* (Han et al., 2012). The unigene TR49263_c0_g1 showed high sequence similarity with *P. ginseng* and *P. quinquefolius*, the highest expression level in root (in the order rhizome_Y > rhizome_O > and secRoot), but had lower expression levels when compared to unigenes within cluster-2 and -3. K-mean clustering showed that all unigenes associated with triterpene saponins biosynthesis were highly expressed in rhizome_Y, rhizome_O and secRoot, and all grouped within cluster-2, and -3. All these unigenes were conserved across three other *Panax* species along with *P. japonicus*, suggesting potential role in saponins biosynthesis. These unigenes, showing similar expression trend as that of those from triterpene saponins biosynthesis pathways would be strong candidates for potential role in ginsenosides biosynthesis.

**FIGURE 8:**
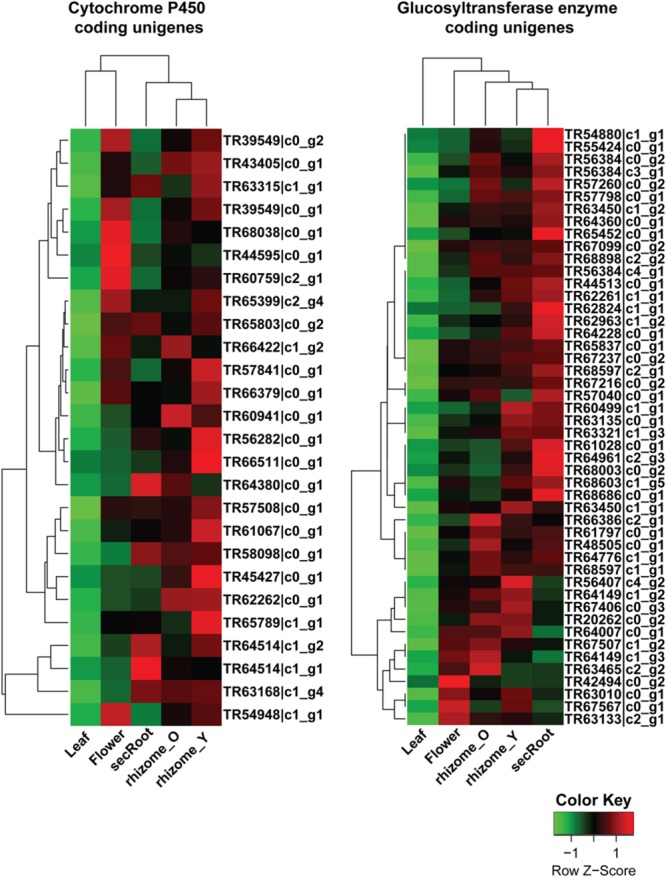
**Expression profile for putative cytochrome P450 (CYP450s) and GTs unigenes from ginsenosides biosynthetic pathways across five tissues of *P. japonicus*.** The expression value (FPKM) for unigenes, annotated as CYP450 or GTs and co-expressed with triterpenoid saponin biosynthetic pathways, were log_2_ transformed and scaled across each row, and heatmap was generated using R-package heatmap2.0.

For cluster-2 and -3, a total of 47 and 74 unigenes were identified being annotated as CYP450 and GTs, respectively. Unigenes with a length of 600 bps or above were selected from these gene clusters, with expression levels across all five tissues shown in the **Figure 8**. Among unigenes annotated as CYP450s, TR45427_c0_g1 showed over 95% of its length aligned with transcripts from all *Panax* species, and was annotated as CYP716A52v2. It has been reported that cytochrome P450 CYP716A52v2 participates in the formation of oleanane-type ginsenosides biosynthesis in *P. ginseng* (Han et al., 2012, 2013). Expression of TR45427_c0_g1 was highest in the rhizome_Y, followed by rhizome_O and secRoot, which was also the case for most of the unigenes from triterpene saponins biosynthesis. Identification of CYP716A52v2 coding unigene among the list of potential P450s involved in the ginsenosides biosynthesis thus validates our approach. Further, expression levels of unigene annotated as CYP716A52v2 were higher than that of unigenes annotated as CYP716A53v2. As CYP716A52v2 is required for oleanane-type ginsenosides, while CYP716A53v2 is required for dammarane-type ginsenosides, therefore, our transcriptome analysis for *P. japonicus* thus supported previous observations suggesting it as oleanane-type ginsenoside rich *Panax* species.

Analyzing unigenes annotated as GTs identified 48 unigenes with a sequence length above 500 bps, and showed high sequence similarity across all *Panax* species studied. Expression trends for these unigenes were different from what we observed for CYP450s. For instance, while all CYP450s and MVA pathway annotated unigenes from cluster-2 and -3 showed high expression in the order of rhizome_Y > followed by rhizome_O > secRoot, unigenes annotated as GTs displayed high expression in the order of secRoot > rhizome_Y > rhizome_O.

## Discussion

Ginsenosides, a pharmacologically active class of metabolites with useful medicinal properties, are the major constituents of root extracts from all 12 plant species from *Panax* genus, with *P. ginseng* and *P. japonicus* being the most popular. Among these *Panax* species, *P. ginseng, P. notoginseng*, and *P. quinquefolius* transcriptome have been studied extensively, while little is known about the transcriptome of *P. japonicus*, which is also considered as the “king of herbs” in the traditional Tujia and Hmong medicine. There are also limited comparative transcriptome studies being done on the *Panax* species, which may provide clues for the identification of conserved metabolic pathways of medicinal importance, particularly for the ginsenosides biosynthesis pathways. Here, we present deep transcriptome profiling and analysis of five tissues of *P. japonicus*, and compared its transcriptome with publically available transcriptome data for three popular *Panax* species, namely, *P. ginseng, P. notoginseng* and *P. quinquefolius*, leading to the identification of 24 CYP450s and 48 GTs as potential genes from the ginsenosides biosynthesis pathways.

*Panax japonicus de novo* transcriptome assembly resulted in 201,660 contigs with a mean length of 873 bps, N50-value as 947 bps, and total number of unigenes as 133,500. Annotation of the *P. japonicus* transcriptome assembly resulted in 67% of its contigs getting a hit against the NCBI-nr database, with the *P. ginseng* as one of the top-hit species. These results taken together suggest that our *de novo* assembly and annotation of transcripts were accurate and consistent with previous reports suggesting *P. japonicus* to be closest to the *P. ginseng*.

Analyzing gene product function using GO annotations for *P. japonicus* transcripts revealed processes related to metabolites oxidation and sugar conjugation being highly enriched. There were 312 unigenes being annotated as CYP450s, and 586 unigenes being assigned to several processes associated with glycosylation process including GTs in the *P. japonicus* transcriptome. For the molecular function categories, several GO categories corresponding to processes, such as UDP-glycosyl transferase activity, hydrolase activity-involving hydrolysing O-glycosyl compounds, oxido-reductase activity-while acting CH-CH groups, and transferase activity-transferring hexoyl groups were assigned to several of the *P. japonicus* transcriptome. Our GO annotation of *P. japonicus* transcriptome, therefore, suggests presence of several transcripts associated with active metabolism, especially in the enzymatic processes involving oxido-reduction reactions, and glycosylation processes, which are also very important for the synthesis of ginsenosides from terpenoids backbone.

Transcripts expression analysis revealed varying number of active transcripts across all five tissues of *P. japonicus*, with secRoot showing highest (69,969), and flower showing lowest (59,228) number of transcripts with non-zero expression value. Our results showed leaf to have the most different transcriptome profile (with approximately 70% of its active transcripts being unique) among rest of the tissues in *P. japonicus*, with rhizome_Y and rhizome_O showing highest number of common transcripts. To further get an insight on GO functional categories enriched with respect to the *P. japonicus* transcriptome assembly in different tissues, we selected unigenes with an expression value (FPKM) above 10 for each tissue to perform GO enrichment analysis. Results diplayed similar GO categories for rhizome_Y, rhizome_O, and secRoot, while leaf showed eight GO categories being specific to this tissue. Enriched GO categories were corroborating with the specific tissues type, and therefore, further validate our transcriptome assembly and annotation. For example, GO enrichment analysis using transcripts with FPKM values above 10 for flower as test set showed GO category corresponding to pollination being enriched and specific only to this tissue, while top four enriched GO categories for leaf included cytosol, generation of precursor metabolites and energy, plastid, and photosynthesis. As we expected, GO category corresponding to photosynthesis was not enriched in the secRoot, rhizome_Y and rhizome_O. Overall, out of the total 53 GO categories combined across five tissues, 25 GO categories were enriched and common across all tissues. This despite of the fact that majority of leaf transcriptome profile were unique, and were not transcriptionally active in the rest of the other tissues. These results thus suggest that while each tissue expressed unique genes, there are processes that are essential for maintaining normal activities of a cell, and therefore, are conserved across all tissues.

In order to identify conserved genes across *Panax* species, we compared *P. japonicus* transcriptome assembly with the transcripts from *P. ginseng, P. notoginseng*, and *P. quinquefolius*. Blastn based sequences alignment across *Panax* species showed high sequence similarity within and with *P. japonicus* transcriptome assembly. For the *P. ginseng*, over 89% of its deposited sequences in the NCBI database showed at least a hit against *P. japonicus* transcriptome assembly, while 83.48% of transcripts from *P. quinquefolius* showed high sequence similarity against *P. japonicus*. Over 65% of *P. japonicus* transcripts showed at least one hit, with 72,223 unique transcripts with no hit against any of the three *Panax* species. Previous studies reported that *P. ginseng* and *P. japonicus* were closest among other *Panax* species, while *P. notoginseng* and *P. quinquefolius* showed high similarity (Choi et al., 2011). Our results in principle does follow these observations. Our study also provided a comprehensive overview of conserved sequences across *Panax* species, which we also used to identify genes associated with ginsenosides biosynthetic pathways.

As triterpenoid saponins biosynthesis pathway is conserved across all *Panax* species, we selected unigenes with sequence length above 500 bps, and having high sequence similarity with all three *Panax* species selected for this study. This approach narrowed down candidate unigenes associated with MVA pathways to 28, and identified all known genes involved in the triterpenoid backbone biosynthesis pathways. All these genes were highly expressed in the rhizome_Y and rhizome_O, followed by secRoot, flower, and leaf, respectively. These results were consistent with previous studies on different *Panax* species, which also observed high expression of MVA associated genes in the roots.

In the ginsenosides biosynthesis, cyclization of 2,3-oxidosqulene, catalyzed by DS (dammarendiol synthase), is the rate limiting step. After the cyclization, the hydroxylation and glycosylation process, which are catalyzed by CYP450s and GTs in turn, are important for the production of triterpene saponins. So far, little is known about different CYP450s and GTs enzymes involved in the ginsenosides biosynthesis, particularly in the *Panax* species. As ginsenosides are derived from the triterpene backbone, we expected co-expression of genes associated with MVA pathways together with genes encoding CYP450s and GTs associated with the ginsenosides biosynthesis pathway. Therefore, we performed k-mean clustering using 28 unigenes associated with the MVA pathway together with 898 unigenes annotated as CYP450s (312 unigenes) or associated with sugar conjugation processes (586), which resulted in six gene clusters. Among these, cluster-2, and -3 included 22 out of 28 unigenes associated with the triterpene backbone synthesis, therefore, genes within these groups were considered as strong candidates to be associated with ginsenosides biosynthesis. Taken together all genes from cluster-2 and -3, resulting in 289 unigenes in total, we identified 24 and 51 unigenes annotated as CYP450 and GTs, respectively, showing high sequence similarity across all the *Panax* species with sequence length above 500 bps. All these unigenes showed high expression in the rhizome_Y and rhizome_O, followed by secRoot, flower, and leaf. As ginsenosides accumulate in the root, these unigenes, therefore, were considered as the potential candidates to be involved in ginsenosides biosynthesis. Our results also suggested that saponins aglycones are synthesized in roots, which is consistent with previous reports on other *Panax* species.

**FIGURE 9:**
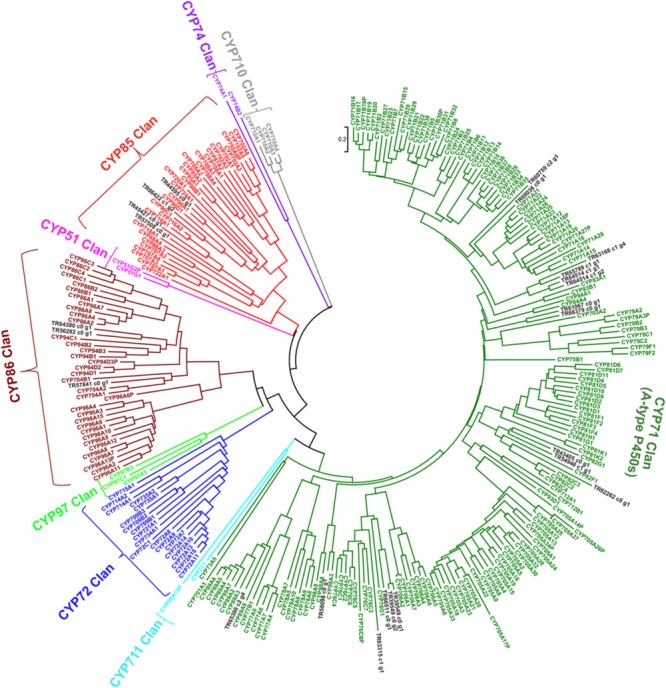
**Phylogenetic analysis of putative CYP450s unigenes from *P. japonicus* transcriptome with all CYP450 genes from *Arabidopsis* genome.** Protein sequences were aligned using MUSCLE program, and evolutionary distances were computed using Jones-Taylor-Thornton (JTT) method. A Neighbor-Joining (NJ) tree was constructed with bootstrap values obtained after 10,000 replications using MEGA6 program.

In order to further characterize CYP450s unigenes identified in this study, we performed phylogenetic analysis for all 24 unigenes together with 244 cytochrome P450 annotated genes in the *Arabidopsis* genome obtained from http://www.p450.kvl.dk/p450.shtml (Paquette et al., 2009), and classified genes in different clades as described (Bak et al., 2011). Majority of unigenes from *P. japonicus* were clustered in CYP71 (17 unigenes) and CYP85 (4 unigenes) clan (**Figure 9**). Previous reports have characterized CYP88D6 from *Glycyrrhiza uralensis* (Seki et al., 2008; CYP85 clan) and CYP93E1 from *Glycine max* (Shibuya et al., 2006; CYP71 clan) to be involved in the triterpene saponins biosynthesis, suggesting CYP450s within these two clan as the most likely candidates to be involved in ginsenosides biosynthesis. Within CYP85 clan, unigene TR45427_co_g1 showed high sequence similarity with all *Panax* species, and was annotated as CYP716A52v2. As *V. vinifera* emerged as the species with highest number of sequences matched to *P. japonicus* transcriptome assembly as top-hit (**Figure 3D**), we performed phylogenetic analysis using these 24 unigenes together with 325 cytochrome P450 genes from *V. vinifera* obtained from http://drnelson.uthsc.edu/vitis.htm, and classified genes in different clades as described (Nelson, 2009). Majority of unigenes were clustered in CYP71 clan (eight Unigenes) with unigene TR45427_co_g1 were grouped in CYP716A clan (**Supplementary Figure S4**). Previous reports have shown that CYP716A52v2 is involved in the ginsenosides biosynthesis. Therefore, identification of CYP716A52v2 annotated unigenes within our set of potential CYP450 candidates using an unbiased approach further indicates that other CYP450 identified in this study will most likely play a role in ginsenosides biosynthesis.

**FIGURE 10:**
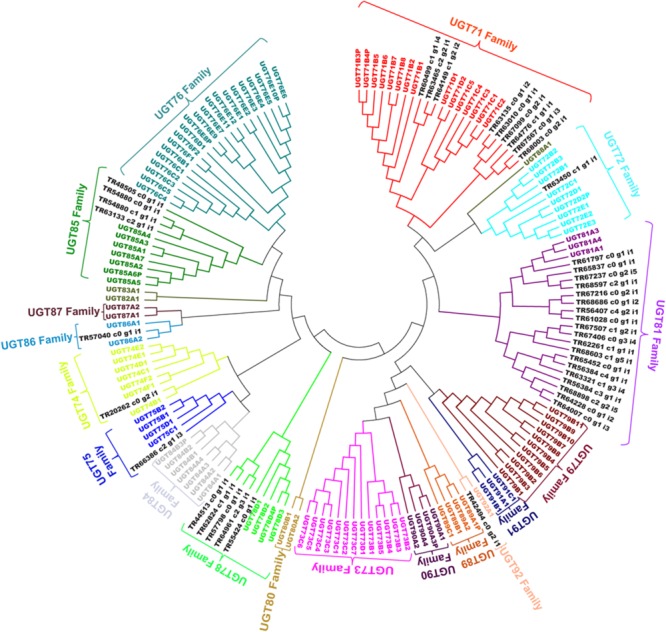
**Phylogenetic analysis of putative GTs unigenes from *P. japonicus* transcriptome with 128 genes annotated as GTs from *Arabidopsis* genome.** Protein sequences were aligned using MUSCLE program, and evolutionary distances were computed using JTT method. A NJ tree was constructed with bootstrap values obtained after 10,000 replications using MEGA6 program.

Expression analysis for unigenes annotated as GTs from cluster-2 and -3 were highly expressed in the roots, with highest expression level in the secRoot, followed by rhizome_Y and rhizome_O, respectively (**Figure 8**). This expression trend was different than what we observed for CYP450s and MVA pathways associated unigenes. Previous reports showed high saponins accumulation in the rhizome of *Panax* species, and roots as the main source of ginsenosides (Yang W.Z. et al., 2014). Our results are consistent with the previous findings, although, our transcriptome data suggested gene expression segregation across different parts of the root. Gene expression trend suggested that while saponins aglycones are synthesized in rhizome_Y and rhizome_O, most likely sugar conjugation occurs in the secRoot. It will be interesting to validate this hypothesis driven from our transcriptome analysis, and to further test if this is due to some transcriptional regulation or translocation of metabolites across these tissues. To further characterize unigenes annotated as GTs, we performed phylogenetic analysis together with 128 genes annotated as GTs from *Arabidopsis* genome, and as classified and obtained from http://www.p450.kvl.dk/p450.shtml (Paquette et al., 2009). Majority of these unigenes were grouped into UGT81 (19 unigenes), UGT71 (9 unigenes) and UGT78 (5 unigenes) families (**Figure 10**). Several genes from *Arabidopsis* within these GTs families have been characterized to conjugate sugar moieties to different classes of metabolites (Benning and Ohta, 2005; Priest et al., 2005; Yonekura-Sakakibara and Hanada, 2011). Our results indicate that these unigenes from *P. japonicus* annotated as GTs are most likely participating in sugar conjugation process of saponin aglycones. Further investigations will be required to ascertain roles of these potential unigenes for their role in ginsenosides biosynthesis.

In summary, *de novo* transcriptome assembly of five tissues of *P. japonicus*, and its comparison with three key *Panax* species revealed great sequence similarity than known before within these major ginsenosides producing plants. Analysis derived from functional annotation and expression analysis can address many biological important questions. We believe that new candidate genes identified as a result of comparative transcriptomics approach will serve as strong candidates for future discoveries on ginsenosides biosynthetic pathways.

## Author Contributions

KS, MY: Conceived and designed the experiments. MN, MK: Performed the experiments. HS: performed deep-transcriptome sequencing. MY, HS: Contributed reagents/materials/analysis tools. AR, MY, HT: Conducted the data interpretation and laboratory analysis. AR, MY, KS: Contributed to the manuscript preparation.

## Conflict of Interest Statement

The authors declare that the research was conducted in the absence of any commercial or financial relationships that could be construed as a potential conflict of interest.

## Footnotes

1 https://www.ncbi.nlm.nih.gov/geo/query/acc.cgi?acc=GSE78893

2 http://www.ncbi.nlm.nih.gov

3 http://pgrc.ipk-gatersleben.de/misa/

4 http://workbench.sdsc.edu/

5 http://medicinalplantgenomics.msu.edu/

## Supplementary Material

FIGURE S1 **Experimental workflow describing experimental design and analysis pipeline used for studying *P. japonicus* transcriptome, and its comparison with other *Panax* species**.

FIGURE S2 **Correlation plot for all five tissues of *P. japonicus* based on expression value of unigenes with non-zero FPKM value.** Unigenes from each tissue with non-zero FPKM expression value were use, and correlation values were calculated and plotted using corrplot from CRAN packages. The color and colored area of the pie chart for each tissue represent *r*^2^-value against rest of the tissues.

FIGURE S3 **Venn diagram for number of unigenes of *P. japonicus* with a blastn hit against *P. ginseng, P. notoginseng*, and *P. quinquefolius* sequences used as blastn search databases**.

FIGURE S4 **Phylogenetic analysis of putative CYP450s unigenes from *P. japonicus* transcriptome with 325 genes annotated as CYP450 from *Vitis vinifera*.** Protein sequences were aligned using MUSCLE program, and evolutionary distances were computed using JTT method.A Neighbor-Joining (NJ) tree was constructed with bootstrap values obtained after 10,000 replications using MEGA6 program.

TABLE S1 **Summary of Trimmomatic program based pre-processing of raw reads for all five tissues of *P. japonicus***.

TABLE S2 **List of KEGG pathways and assigned unigenes from the *P. japonicus* transcriptome assembly**.

TABLE S3 **Identified SSRs motifs and its frequency in the *P. japonicus* transcriptome assembly**.

TABLE S4 **Transcriptome data source for *P. ginseng, P. notoginseng*, and *P. quinquefolius* to perform comparative analysis with the *P. japonicus* transcriptome assembly**.

TABLE S5 **Unigenes from the *P. japonicus* transcriptome assembly grouped in six different gene clusters by k-mean clustering analysis**.
