# Supplementary figures and images for: RNA-seq Transcriptome Analysis of Panax japonicus, and Its Comparison with Other Panax Species to Identify Potential Genes Involved in the Saponins Biosynthesis
Source: Front Plant Sci. 2016 Apr 12;7:481. doi: 10.3389/fpls.2016.00481 (PMC4828455; doi:10.3389/fpls.2016.00481)

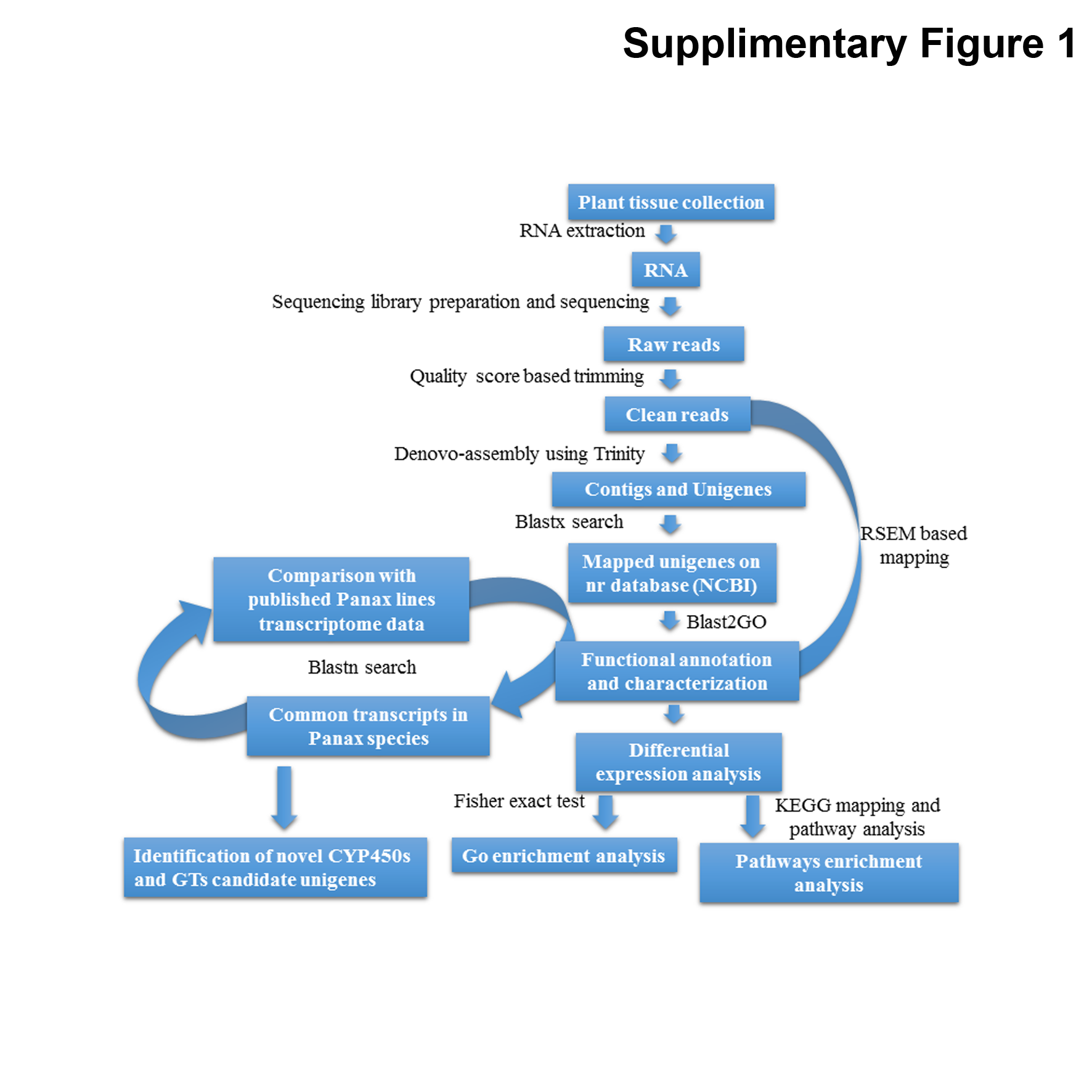

Supplement: FIGURE S1 — Experimental workflow describing experimental design and analysis pipeline used for studying P. japonicus transcriptome, and its comparison with other Panax species. [file Image_1.TIF]

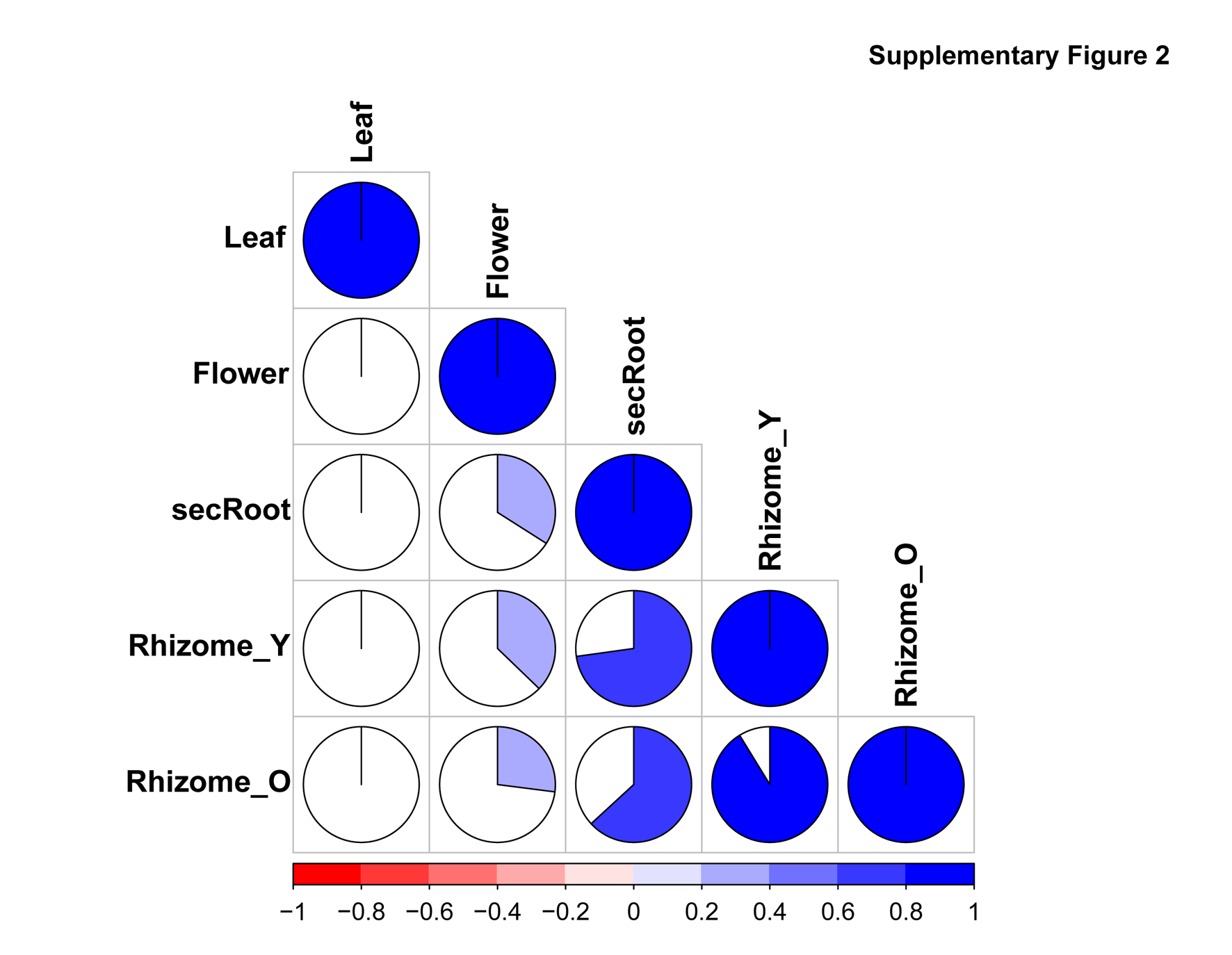

Supplement: FIGURE S2 — Correlation plot for all five tissues of P. japonicus based on expression value of unigenes with non-zero FPKM value. Unigenes from each tissue with non-zero FPKM expression value were use, and correlation values were calculated and plotted using corrplot from CRAN packages. The color and colored area of the pie chart for each tissue represent r2-value against rest of the tissues. [file Image_2.TIF]

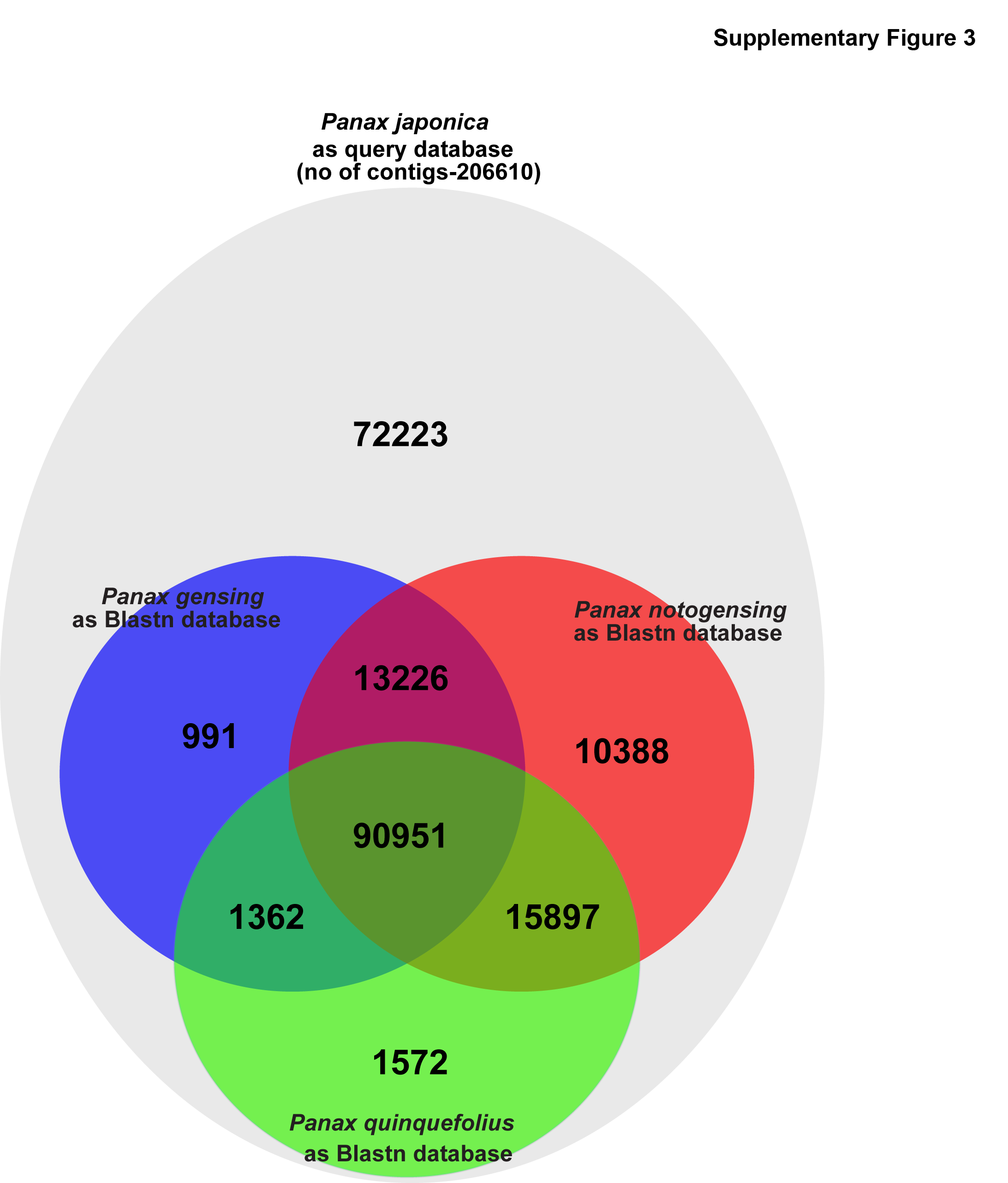

Supplement: FIGURE S3 — Venn diagram for number of unigenes of P. japonicus with a blastn hit against P. ginseng, P. notoginseng, and P. quinquefolius sequences used as blastn search databases. [file Image_3.TIF]

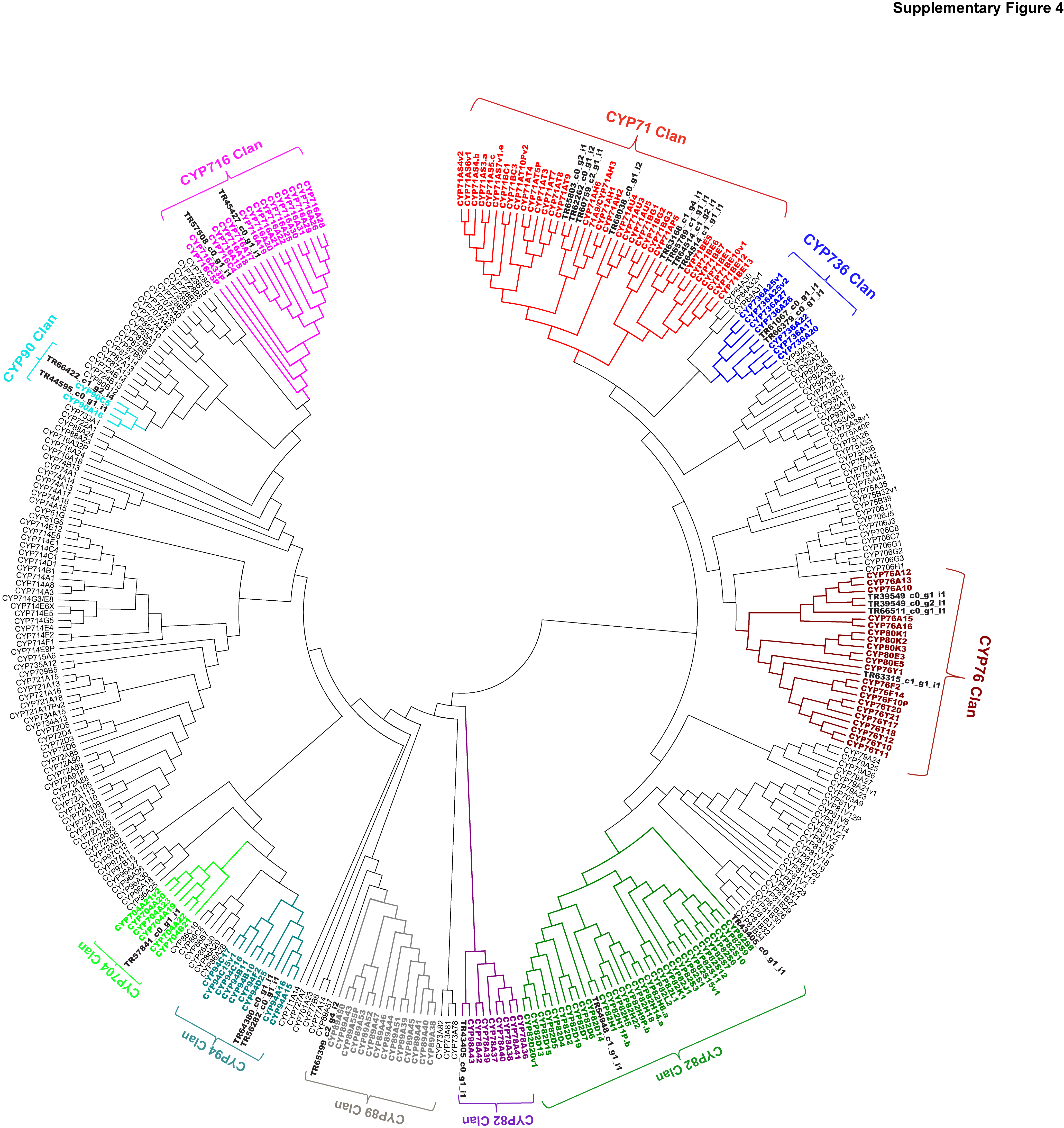

Supplement: FIGURE S4 — Phylogenetic analysis of putative CYP450s unigenes from P. japonicus transcriptome with 325 genes annotated as CYP450 from Vitis vinifera. Protein sequences were aligned using MUSCLE program, and evolutionary distances were computed using JTT method.A Neighbor-Joining (NJ) tree was constructed with bootstrap values obtained after 10,000 replications using MEGA6 program. [file Image_4.TIF]
