# Supplementary material for: RNA-seq Transcriptome Analysis of Panax japonicus, and Its Comparison with Other Panax Species to Identify Potential Genes Involved in the Saponins Biosynthesis
Source: Front Plant Sci. 2016 Apr 12;7:481. doi: 10.3389/fpls.2016.00481 (PMC4828455; doi:10.3389/fpls.2016.00481)
Supplement: TABLE S1 — Summary of Trimmomatic program based pre-processing of raw reads for all five tissues of P. japonicus. [file Table_1.DOCX]

| **Supplementary Table 1- Summary of Trimmomatic program based pre-processing of raw reads for all five tissues of *P. japonicus***   \|  \| **Flower** \| **secRoot** \| **Rhizome_Y** \| **Rhizome_O** \| **Leaf** \| \| --- \| --- \| --- \| --- \| --- \| --- \| \| **Input pair reads** \| 3,185,218 \| 6,431,034 \| 3,692,881 \| 4,445,241 \| 6,174,181 \| \| **Both Surviving** \| 2,979,595 (93.54%) \| 6,027,845 (93.73%) \| 3,473,960 (94.07%) \| 4,156,821 (93.51%) \| 5,842,475 (94.63%) \| \| **Forward Only Surviving** \| 195,553 (6.14%) \| 383,261 (5.96%) \| 207,642 (5.62%) \| 274,707 (6.18%) \| 312,185 (5.06%) \| \| **Reverse Only Surviving** \| 5,497 (0.17%) \| 10,472 (0.16%) \| 6,351 (0.17%) \| 7,344 (0.17%) \| 10,760 (0.17%) \| \| **Dropped** \| 4,573 (0.14%) \| 9,456 (0.15%) \| 4,928 (0.13%) \| 6,369 (0.14%) \| 8,761 (0.14%) \| |  |  |  |  |  |  |  |  |  |
| --- | --- | --- | --- | --- | --- | --- | --- | --- | --- | --- | --- | --- | --- | --- | --- | --- | --- | --- | --- | --- | --- | --- | --- | --- | --- | --- | --- | --- | --- | --- | --- | --- | --- | --- | --- | --- | --- | --- | --- | --- | --- | --- | --- | --- | --- |
|  |  |  |  |  |  |  |  |  |  |
|  |  |  |  |  |  |  |  |  |  |
|  |  |  |  |  |  |  |  |  |  |
|  |  |  |  |  |  |  |  |  |  |
|  |  |  |  |  |  |  |  |  |  |
