# Supplementary material for: RNA-seq Transcriptome Analysis of Panax japonicus, and Its Comparison with Other Panax Species to Identify Potential Genes Involved in the Saponins Biosynthesis
Source: Front Plant Sci. 2016 Apr 12;7:481. doi: 10.3389/fpls.2016.00481 (PMC4828455; doi:10.3389/fpls.2016.00481)
Supplement: TABLE S4 — Transcriptome data source for P. ginseng, P. notoginseng, and P. quinquefolius to perform comparative analysis with the P. japonicus transcriptome assembly. [file Table_4.DOCX]

**Supplementary Table 4**- Transcriptome data source for *P. ginseng*, *P. notoginseng*, and *P. quinquefolius* to perform comparative analysis with *P. japonicus* transcriptome assembly.

| Panax species | Source | Assembly approach | Total no of contigs | Min length | Max length | N20 | N50 | N80 |
| --- | --- | --- | --- | --- | --- | --- | --- | --- |
| Panax notogensing | \| SRR1032053,  SRR1032061,  SRR1032062,  SRR1562019,  SRR1562020,  SRR1562021,  SRR1562022,  SRR1562023,  SRR1562024,  SRR1562025,  SRR1562026, \|  \|  \| \| --- \| --- \| --- \| \|  \|  \| \|  \|  \| \|  \|  \| \|  \|  \| \|  \|  \| \|  \|  \| \|  \|  \| \|  \|  \| \|  \|  \| \|  \|  \| | Trinity | 254971 | 224 | 27485 | 1697 | 735 | 348 |
| Panax gensing | NCBI EST database | Not applicable | 106320 | 224 | 464680 | 1225 | 742 | 463 |
| Panax quinquefolius | Medicinal Plant Genomics Resource  (pqa_assembly_v_10072011.fasta) | Not applicable | 110795 | 201 | 11566 | 2517 | 1475 | 668 |
